# Supplementary material for: Analysis of Lsm Protein-Mediated Regulation in the Haloarchaeon Haloferax mediterranei
Source: Int J Mol Sci. 2024 Jan 1;25(1):580. doi: 10.3390/ijms25010580 (PMC10779274; doi:10.3390/ijms25010580)
Supplement: Supplementary file 1 [file ijms-25-00580-s001.zip › Figure S1_mod.pdf]

Alignment model 1: reference sequence *Hbt. salinarum*

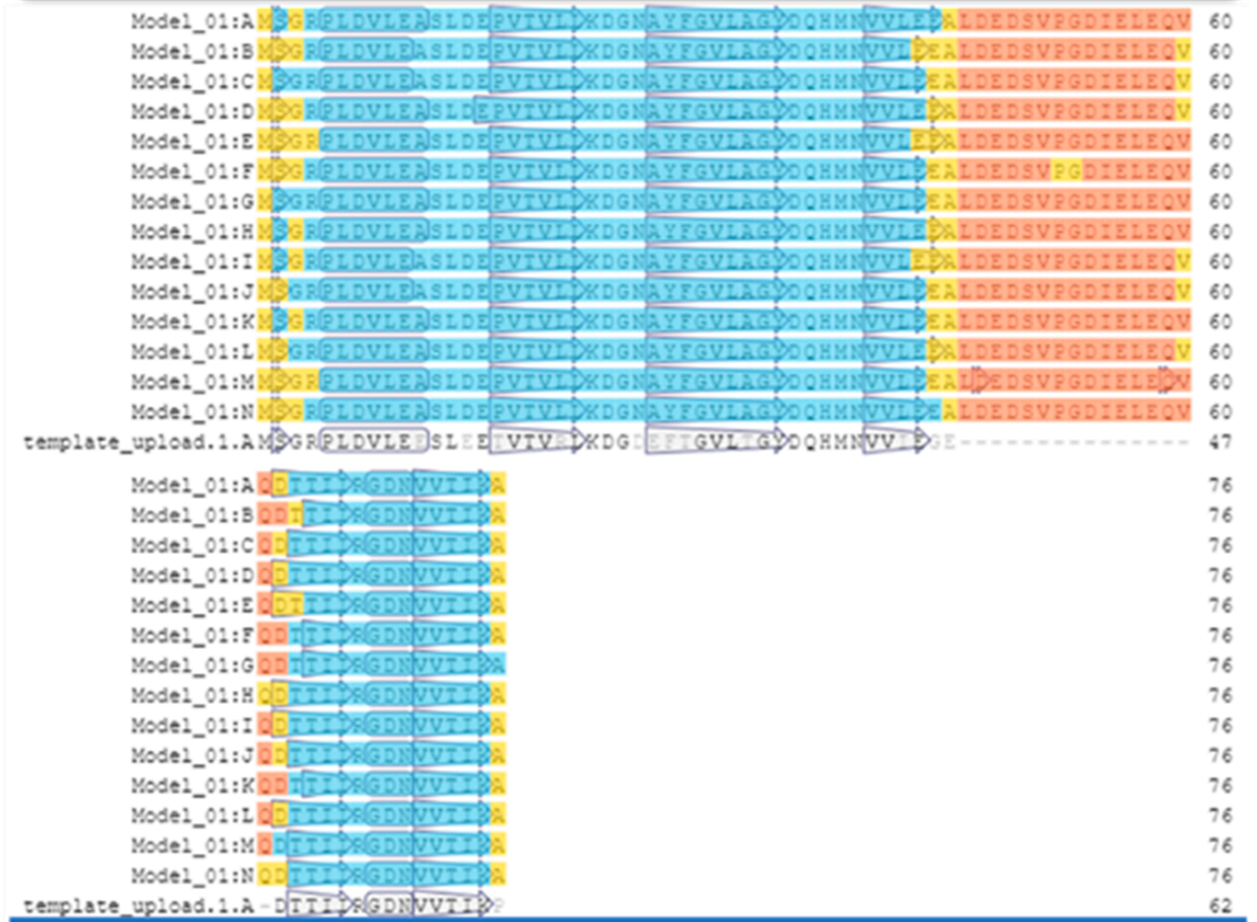

Alignment model 2: reference sequence *A.fulgidus*

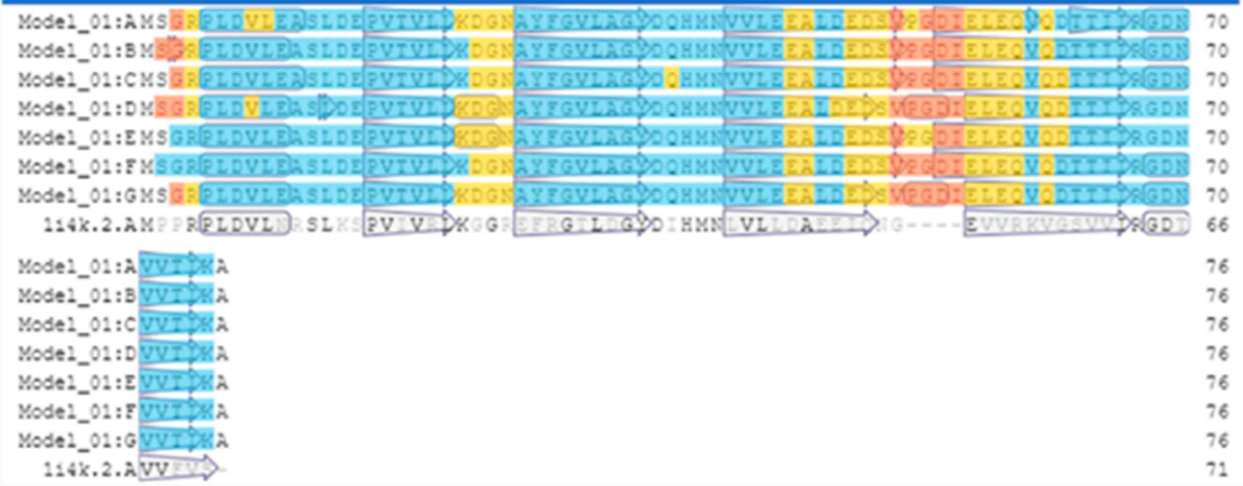

Alignment model 3: reference sequence *P.abysii*

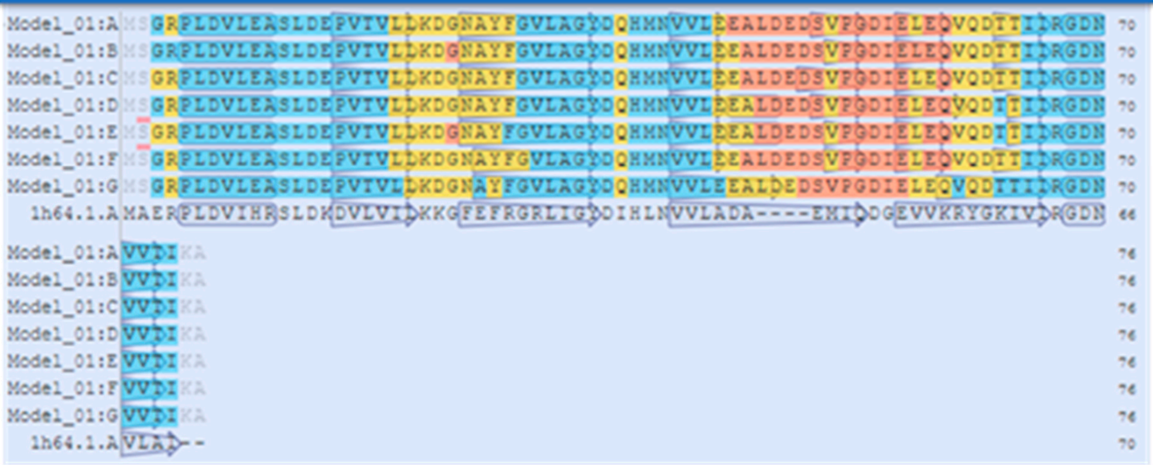

Figure S1. Alignments obtained for modeling through homology (SWISS-MODEL from Expasy). The blue residues have a high confidence index (>0.85), the yellow residues have a medium confidence index (0.85-0.5) and the orange residues have a lower confidence index (<0.5).
