# Supplementary material for: Analysis of Lsm Protein-Mediated Regulation in the Haloarchaeon Haloferax mediterranei
Source: Int J Mol Sci. 2024 Jan 1;25(1):580. doi: 10.3390/ijms25010580 (PMC10779274; doi:10.3390/ijms25010580)
Supplement: Supplementary file 1 [file ijms-25-00580-s001.zip › Figure S3_mod.pdf]

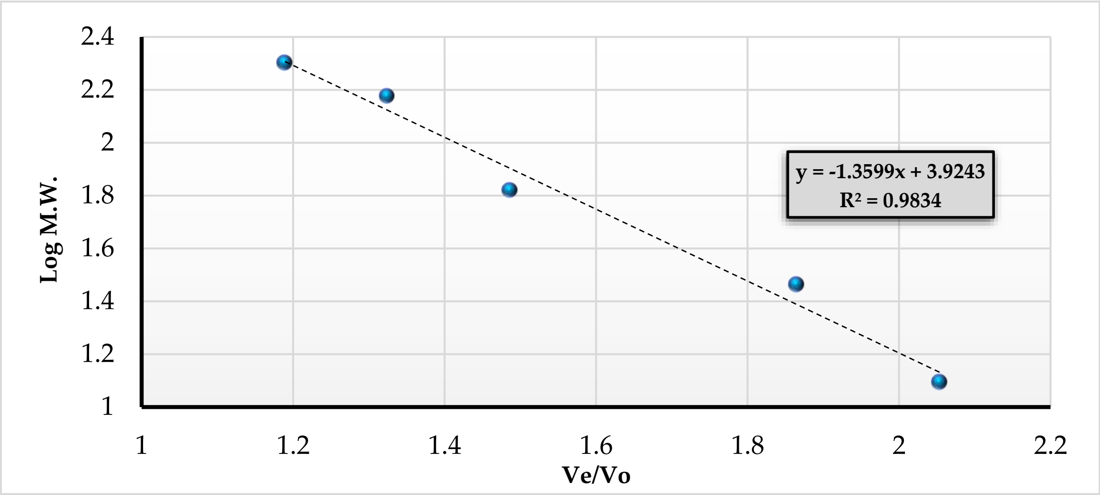

Figure S3. Calibration curve of standards of known molecular weight. The protein standards used were Gel Filtration Molecular Weight Markers Kit for Molecular Weights 12,000–200,000 Da (SigmaAldrich). Dextran blue (2000 kDa); β-amylase (200 kDa); Alcohol dehydrogenase (150 kDa); Albumin (66 kDa); Carbonic anhydrase (29 kDa); Cytochrome c (12.4 kDa).
