# Supplementary material for: Analysis of Lsm Protein-Mediated Regulation in the Haloarchaeon Haloferax mediterranei
Source: Int J Mol Sci. 2024 Jan 1;25(1):580. doi: 10.3390/ijms25010580 (PMC10779274; doi:10.3390/ijms25010580)
Supplement: Supplementary file 1 [file ijms-25-00580-s001.zip › lsm_AlphaFold2-models.pptx]

## Slide 1
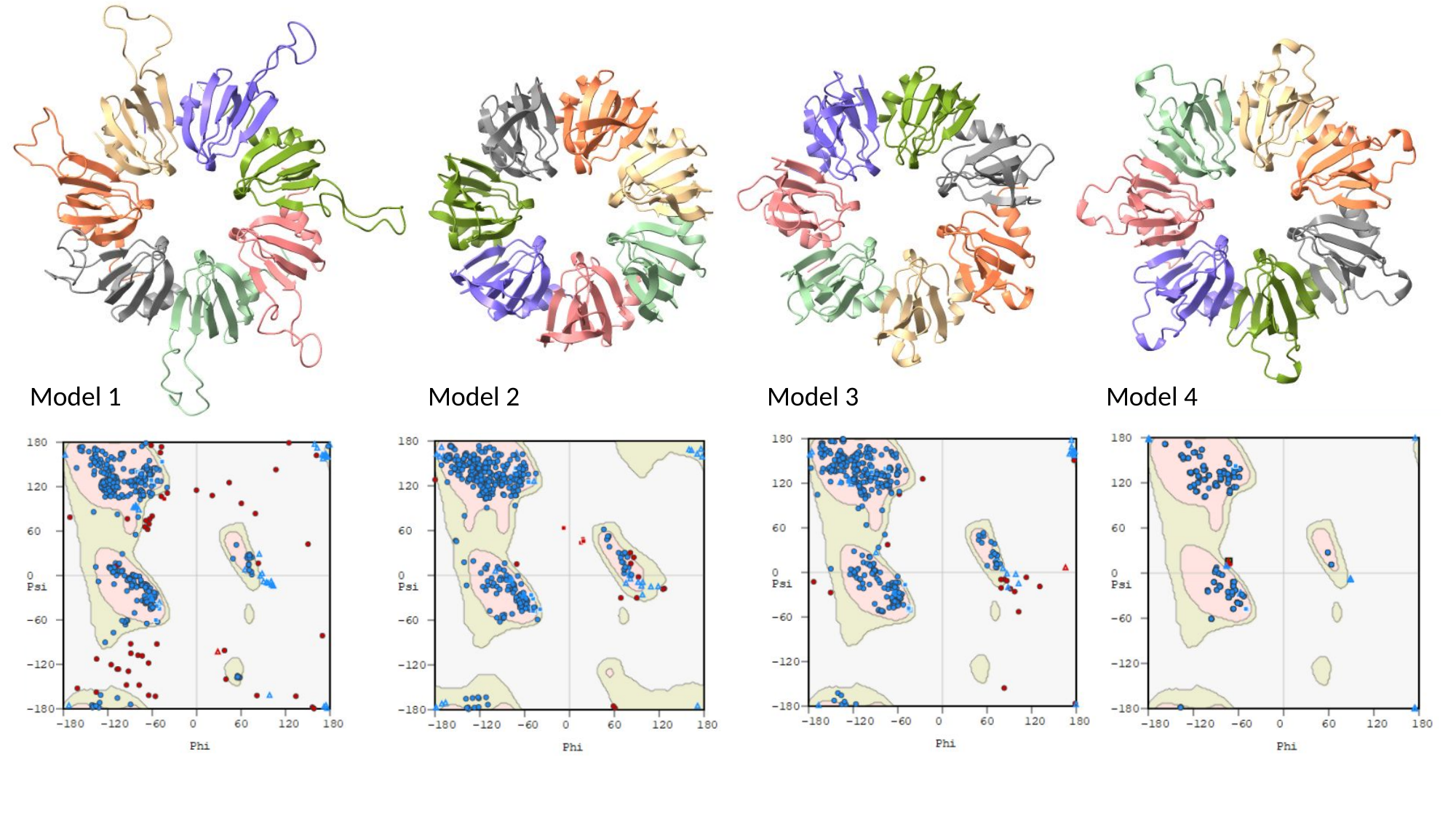

Model 1
Model 2
Model 3
Model 4

## Slide 2
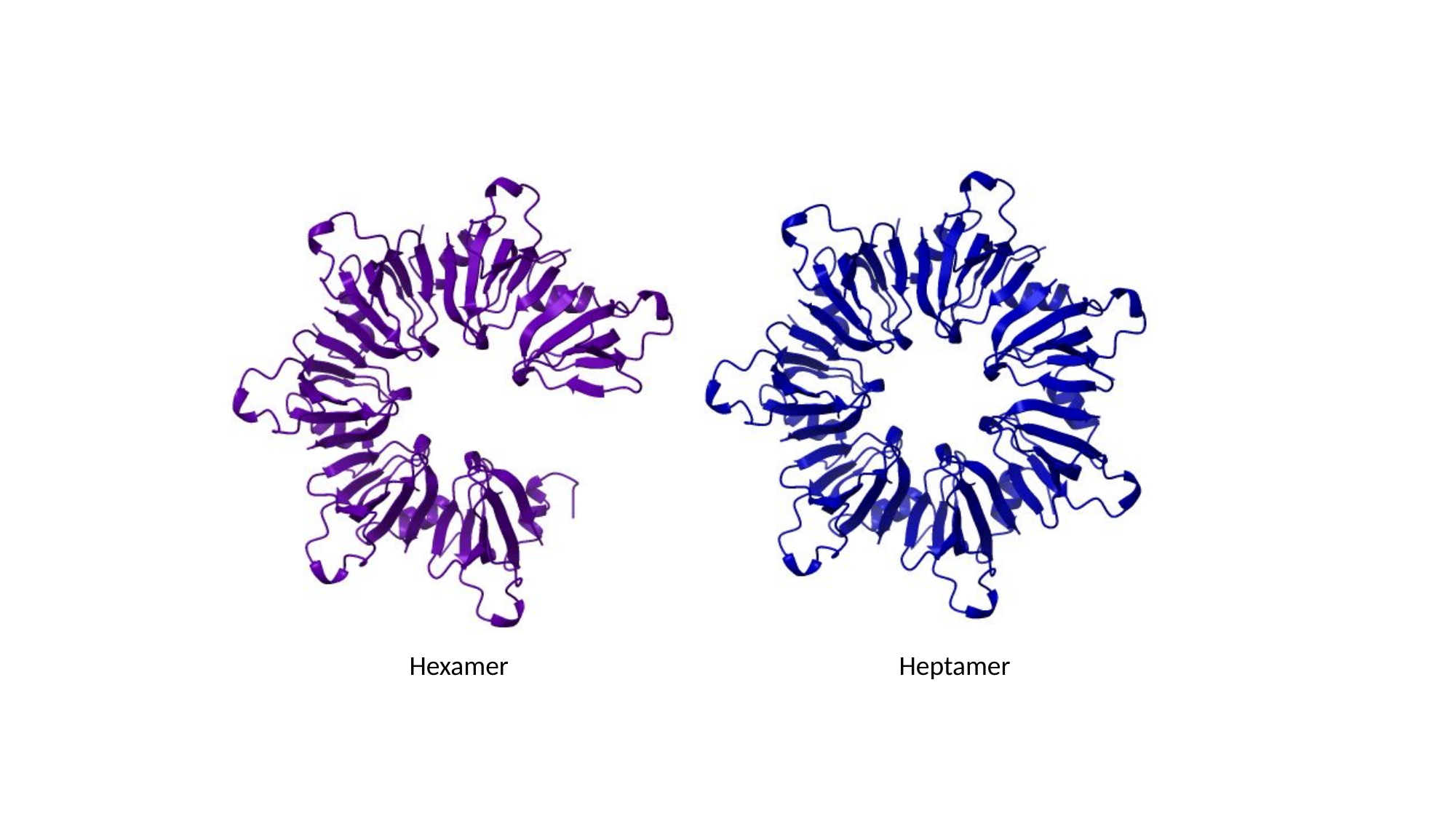

Hexamer
Heptamer

## Slide 3
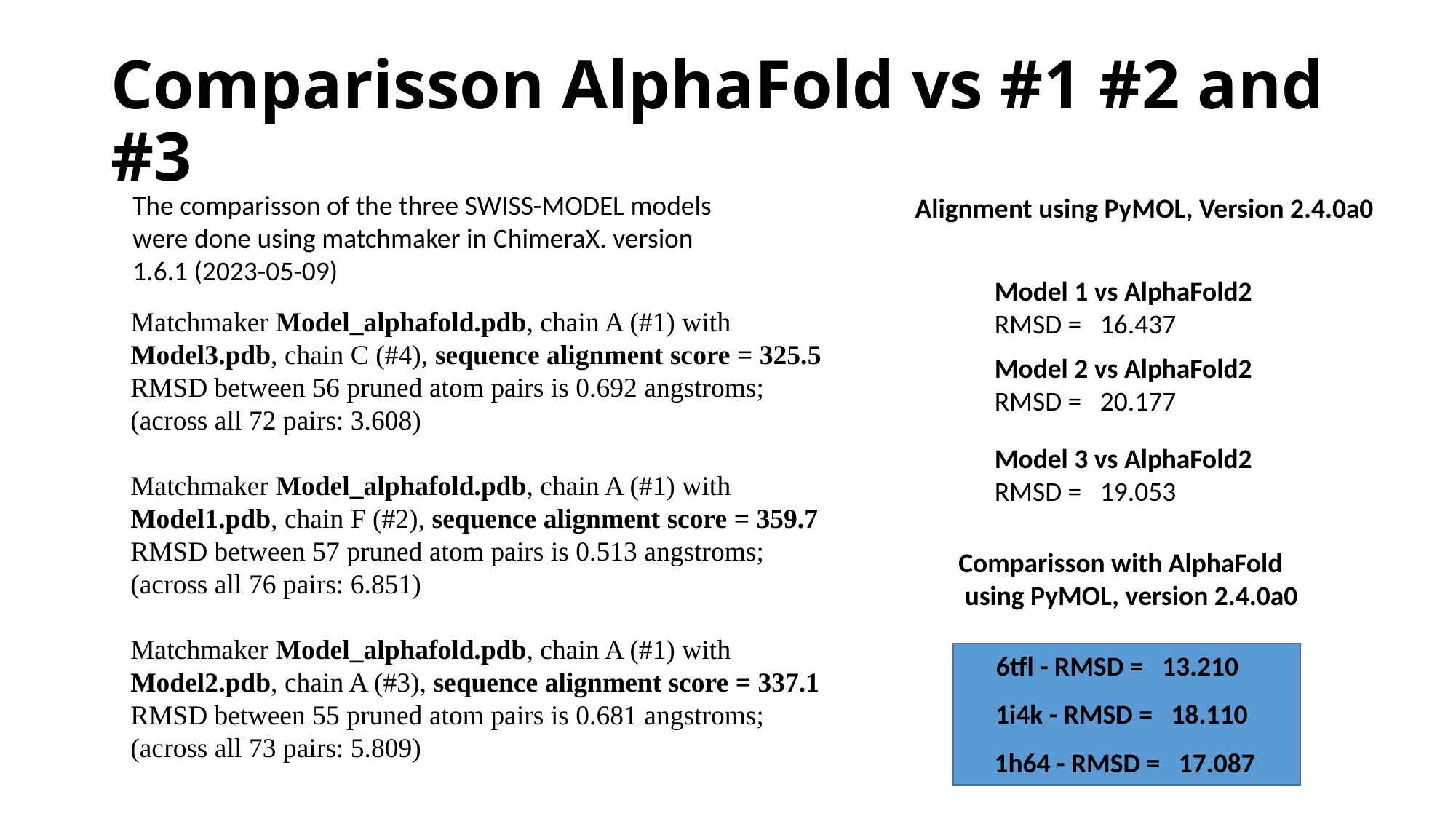

# Comparisson AlphaFold vs #1 #2 and #3
The comparisson of the three SWISS-MODEL models were done using matchmaker in ChimeraX. version 1.6.1 (2023-05-09)
Alignment using PyMOL, Version 2.4.0a0
Model 1 vs AlphaFold2
RMSD = 16.437
Matchmaker Model_alphafold.pdb, chain A (#1) with Model3.pdb, chain C (#4), sequence alignment score = 325.5
RMSD between 56 pruned atom pairs is 0.692 angstroms; (across all 72 pairs: 3.608)
Matchmaker Model_alphafold.pdb, chain A (#1) with Model1.pdb, chain F (#2), sequence alignment score = 359.7
RMSD between 57 pruned atom pairs is 0.513 angstroms; (across all 76 pairs: 6.851)
Matchmaker Model_alphafold.pdb, chain A (#1) with Model2.pdb, chain A (#3), sequence alignment score = 337.1
RMSD between 55 pruned atom pairs is 0.681 angstroms; (across all 73 pairs: 5.809)
Model 2 vs AlphaFold2
RMSD = 20.177
Model 3 vs AlphaFold2
RMSD = 19.053
Comparisson with AlphaFold
 using PyMOL, version 2.4.0a0
6tfl - RMSD = 13.210
1i4k - RMSD = 18.110
1h64 - RMSD = 17.087

## Slide 4
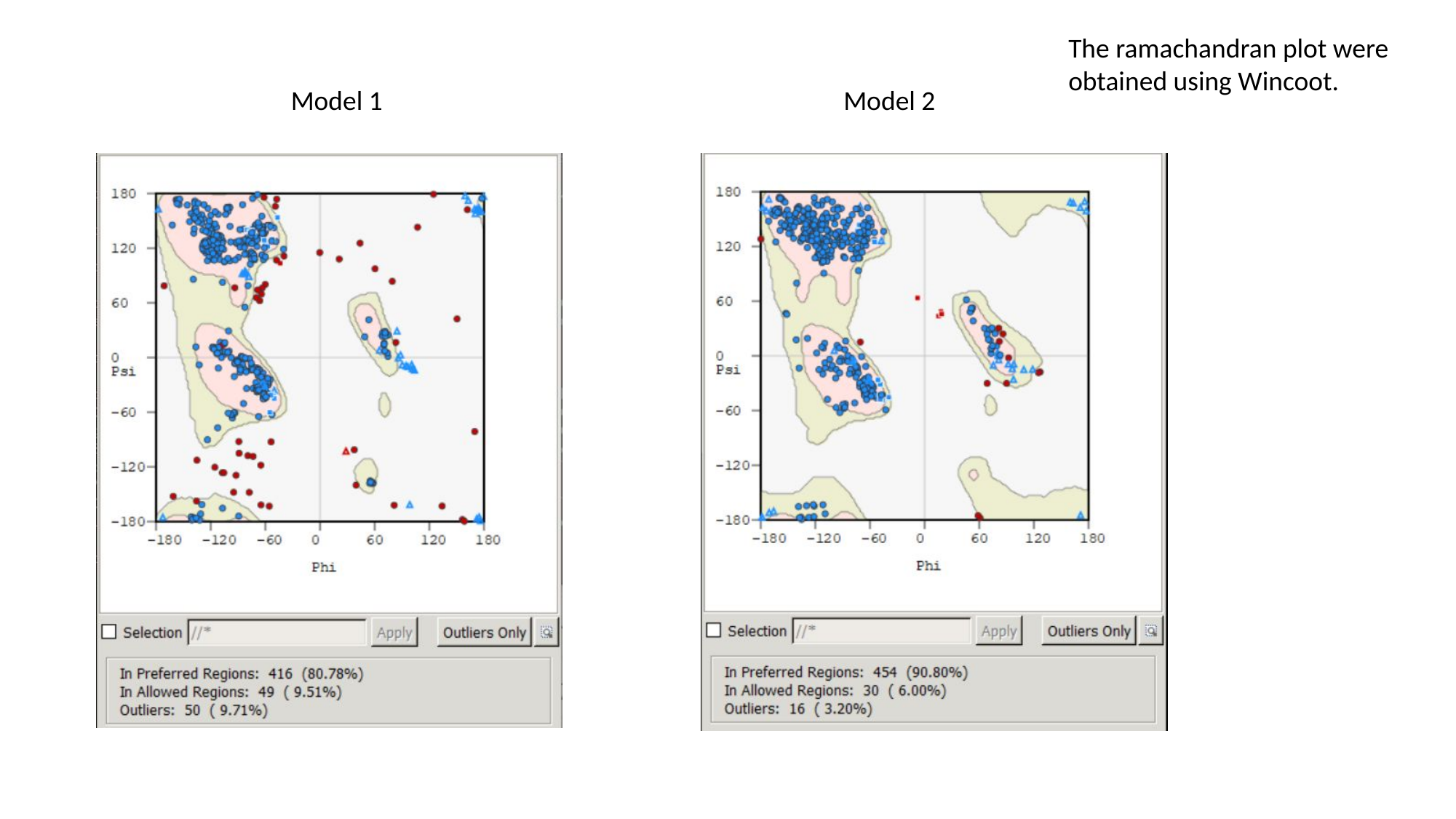

The ramachandran plot were obtained using Wincoot.
Model 1
Model 2

## Slide 5
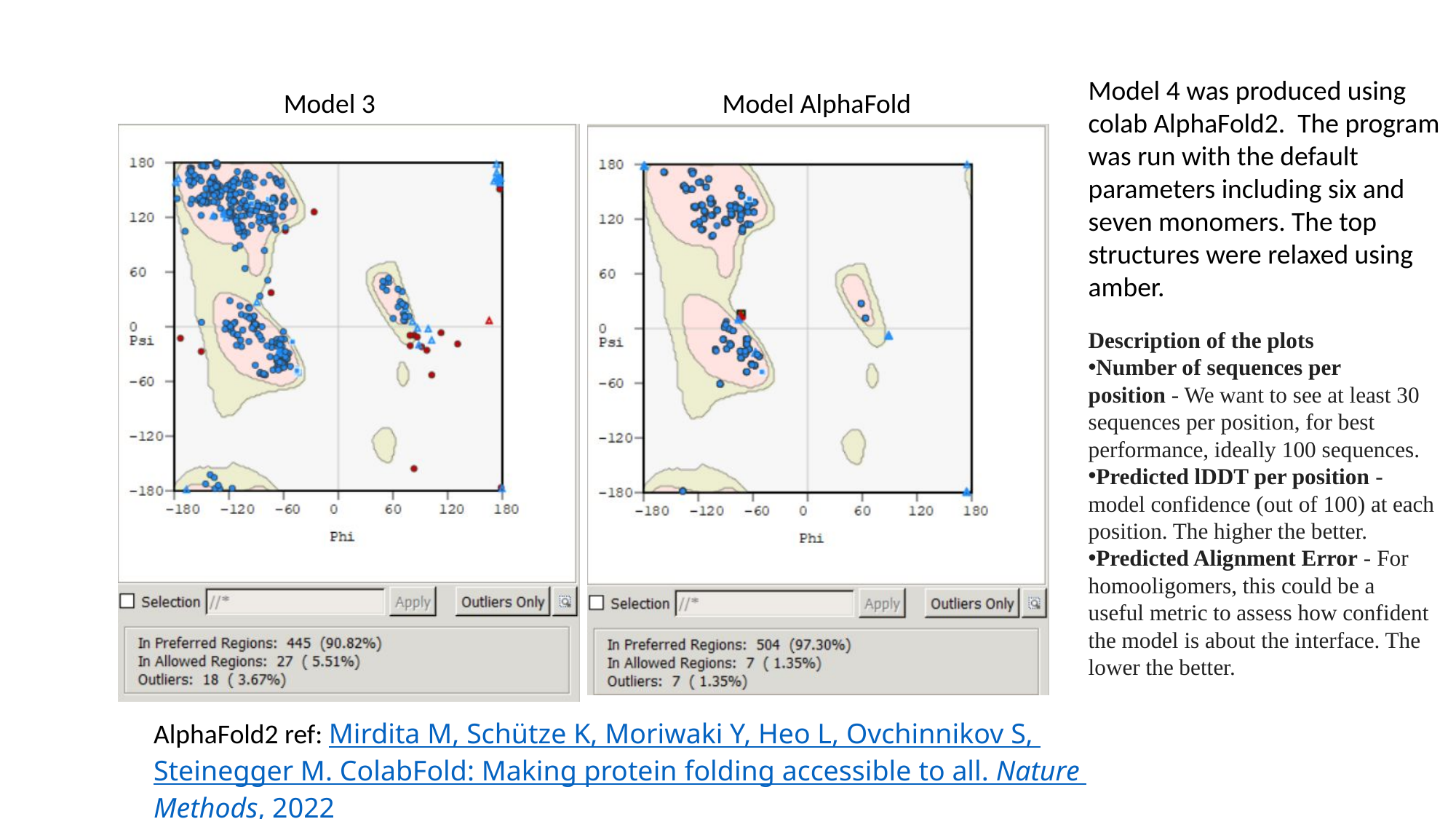

Model 4 was produced using colab AlphaFold2. The program was run with the default parameters including six and seven monomers. The top structures were relaxed using amber.
Model 3
Model AlphaFold
Description of the plots
Number of sequences per position - We want to see at least 30 sequences per position, for best performance, ideally 100 sequences.
Predicted lDDT per position - model confidence (out of 100) at each position. The higher the better.
Predicted Alignment Error - For homooligomers, this could be a useful metric to assess how confident the model is about the interface. The lower the better.
AlphaFold2 ref: Mirdita M, Schütze K, Moriwaki Y, Heo L, Ovchinnikov S, Steinegger M. ColabFold: Making protein folding accessible to all. Nature Methods, 2022

## Slide 6
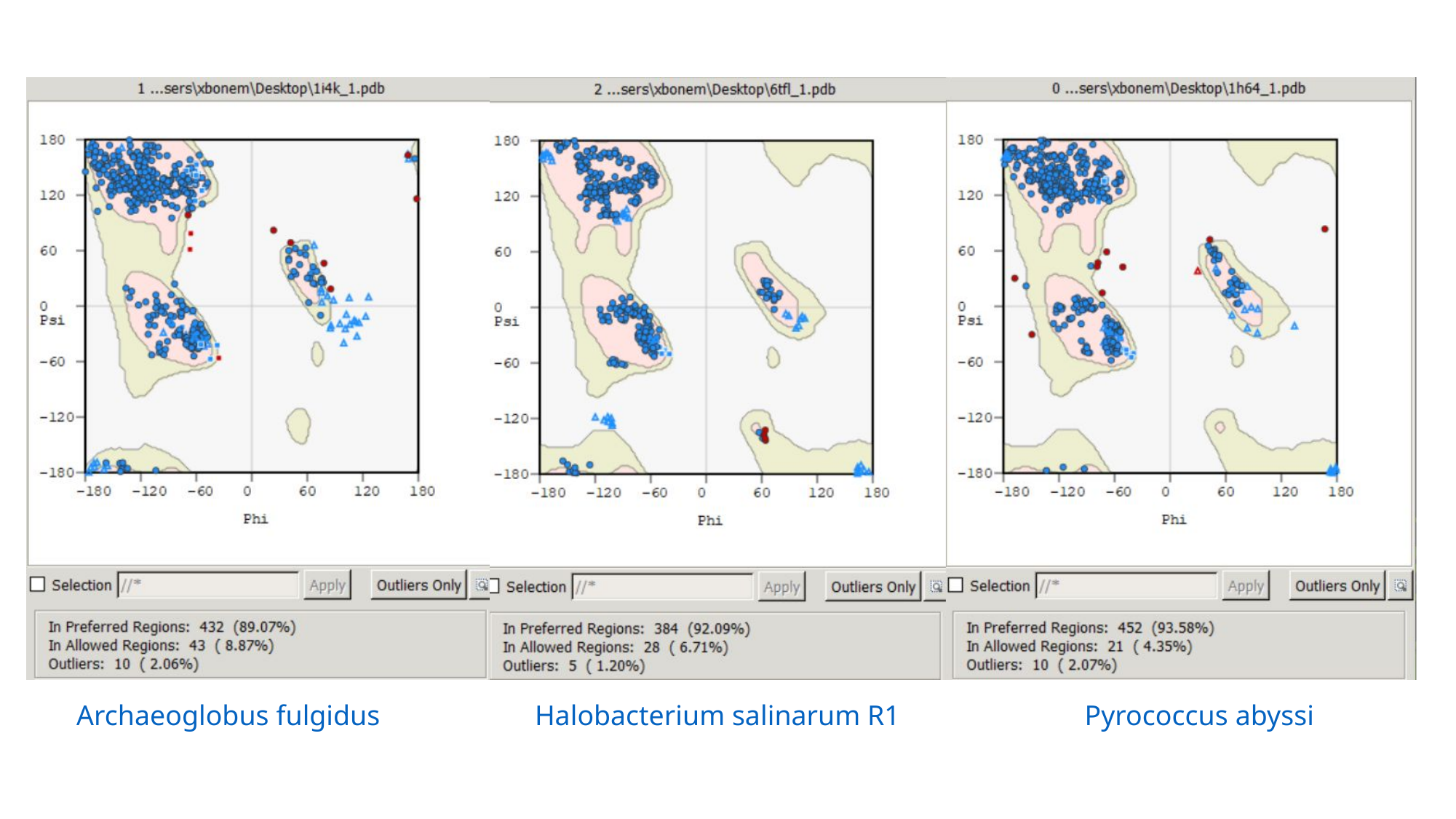

Archaeoglobus fulgidus
Halobacterium salinarum R1
Pyrococcus abyssi
