# Supplementary material for: Analysis of Lsm Protein-Mediated Regulation in the Haloarchaeon Haloferax mediterranei
Source: Int J Mol Sci. 2024 Jan 1;25(1):580. doi: 10.3390/ijms25010580 (PMC10779274; doi:10.3390/ijms25010580)
Supplement: Supplementary file 1 [file ijms-25-00580-s001.zip › Table S1.pdf]

Table S1. Modelling results using SWISS-Model (Expasy). GMQE: measures model quality dependent on coverage; QMEAN: measures model quality independent of coverage.

	**MODEL 1**	**MODEL 2**	**MODEL 3**
**Reference protein**	6TFL_A	1I4K_1	1H64_1
**Reference species**	*Hbt. salinarum*	*A. fulgidus*	*P. abyssi*
**Sequence identity (%)**	78.33	46.48	48.57
**Sequence similarity**	0.53	0.40	0.41
**Coverage**	0.79	0.93	0.92
**Biological unit**	Homo-14-Mer	Homoheptamer	Homoheptamer
**Ligand**	14 x Uridines	-	-
**GMQE**	0.65	**0.74**	0.66
**QMEAN**	0.71	**0.73**	0.68
