# Supplementary material for: Analysis of Lsm Protein-Mediated Regulation in the Haloarchaeon Haloferax mediterranei
Source: Int J Mol Sci. 2024 Jan 1;25(1):580. doi: 10.3390/ijms25010580 (PMC10779274; doi:10.3390/ijms25010580)
Supplement: Supplementary file 1 [file ijms-25-00580-s001.zip › Table S2.pdf]

Table S2. Results obtained in the Ramachandran diagram of each of the Lsm protein models of *Hfx. mediterranei* using MolProbity.

	MODEL 1	MODEL 2	MODEL 3	MODEL 4
**Reference microorganism**	*Hbt. salinarum*	*A. fulgidus*	*P. abyssi*	Alphafold
**Preferred regions**	80.78 %	90.80 %	90.82 %	97.30 %
**Allowed regions**	9.51 %	6.00 %	5.51 %	1.35 %
**Outliers**	9.71 %	3.20 %	3.67 %	1.35 %
