# Supplementary material for: Analysis of Lsm Protein-Mediated Regulation in the Haloarchaeon Haloferax mediterranei
Source: Int J Mol Sci. 2024 Jan 1;25(1):580. doi: 10.3390/ijms25010580 (PMC10779274; doi:10.3390/ijms25010580)
Supplement: Supplementary file 1 [file ijms-25-00580-s001.zip › Table S3.pdf]

Table S3. List of up-expressed and down-expressed genes in the HM26-Δ*Sm1* versus HM26 contrast under carbon starvation.

Locus	Name	Metabolism	Log_2FC
**Genes *up-expressed***			
HFX_6257	Type IV pilin	Signaling and cellular processes: cell motility	2.98
HFX_4107	C2H2-type zinc finger protein	gene expression	2.40
HFX_0688	PadR family transcriptional regulator	Gene expression	2.37
**Genes *down-expressed***			
HFX_5079	Biotin synthase BioB	Cofactors and vitamins: biotin synthesis	-1.98
HFX_1377	CBS domain-containing protein		-2.00
HFX_6051	Thiolase family protein	- Carbon metabolism<br>- Amino acid degradation (Trp, Val, Leu, Ile, Lys)<br>- Fatty acid degradation<br>- Degradation of ketone bodies<br>- Benzoate degradation<br>- Biosynthesis of secondary metabolites	-2.16
HFX_6278	High-potential iron-sulfur protein	- Energy metabolism	-2.56
HFX_6411	Twin-arginine translocation signal domain-containing protein	- Transport processes	-3.92
