# Supplementary material for: Analysis of Lsm Protein-Mediated Regulation in the Haloarchaeon Haloferax mediterranei
Source: Int J Mol Sci. 2024 Jan 1;25(1):580. doi: 10.3390/ijms25010580 (PMC10779274; doi:10.3390/ijms25010580)
Supplement: Supplementary file 1 [file ijms-25-00580-s001.zip › Table S4.pdf]

Table S4. List of up-expressed and down-expressed genes in the HM26-Δ*Sm1* versus HM26 contrast under nitrogen starvation.

Locus	Name	Metabolism	Log2FC
**Genes up-expressed**			
**HFX_4025**	Alkyl sulfatase dimerization domain-containing protein		2.77
**HFX_4023**	Aldehyde dehydrogenase family protein	• Carbon metabolism (Glycolysis/ Gluconeogenesis; pyruvate metabolism)<br>• Fatty acid degradation<br>• Amino acid degradation (valine, leucine, isoleucine and lysine)<br>• Glycerolipid metabolism<br>• Biosynthesis of secondary metabolites and cofactors	2.58
**HFX_1552**	Acetamidase/formamidase family protein		2.33
**HFX_1281**	Beta-ketoacyl-ACP reductase	• Fatty acid biosynthesis<br>• Biotin biosynthesis<br>• Biosynthesis of secondary metabo-lites	2.22
**HFX_1908**	SHOCT domain-containing protein		2.01

Table S4 continued. List of up-expressed and down-expressed genes in the contrast HM26Δ*Sm1* versus HM26 in the absence of a nitrogen source.

Genes down-expressed			
HFX_0944	Cytochrome c oxidase subunit I	Energy metabolism	-2.07
HFX_0942	ba3-type terminal oxidase subunit CbaD		-2.13
HFX_1924	4Fe-4S dicluster domain-containing protein		-2.25
HFX_0429	Cytochrome ubiquinol oxidase subunit I		-2.34
HFX_0721	DoxX family membrane protein (thiosulfate dehydrogenase (quinone) large subunit		-2.34
HFX_1925	FAD-dependent oxidoreductase		-2.55
HFX_0428	Cytochrome d ubiquinol oxidase subunit II		-2.72
HFX_2214	molybdopterin-dependent oxidoreductase		-2.78
HFX_0943	Cytochrome c oxidase subunit II		-2.82
HFX_6278	High-potential iron-sulfur protein		-3.01
HFX_1927	Electron transfer flavoprotein subunit beta		-3.14
HFX_6343	DoxX family membrane protein (thiosulfate dehydrogenase (quinone) large subunit		-3.17
HFX_1926	Electron transfer flavoprotein subunit alpha		-3.33
HFX_1958	Aldo/keto reductase	Carbon metabolism	-2.12
HFX_0946	Universal stress protein	Environmental information processing under stressful conditions	-2.05
HFX_1094	Universal stress protein		-2.09
HFX_2288	Universal stress protein		-2.13
HFX_1885	Universal stress protein		-3.10
HFX_1928	Universal stress protein		-3.24
HFX_5076	BioB dethiobiotin synthase	Metabolism of cofactors and vitamins	-2.22
HFX_5079	BioB biotin synthase BioB		-2.66
HFX_5077	8-amino-7- oxononanoate synthase		-2.73
HFX_1129	radical SAM/SPASM domain-containing protein (heme biosynthesis protein)		-2.30
HFX_1341	helix-turn-helix domain-containing protein	Gene expression	-2.06
HFX_0165	Winged helix-turn-helix transcriptional regulator		-2.42
HFX_5091	TAT-dependent nitrous oxide reductase	• Nitrogen metabolism<br>• Energy metabolism	-2.19
HFX_1359	Molybdenum transporter	Transport	-2.26
HFX_1377	CBS domain-containing protein	-	-2.59
HFX_5231	CBS domain-containing protein		-3.07
HFX_5244	DUF11 domain-containing protein		-2.79
HFX_6361	MaoC/PaaZ C-terminal domain-containing protein		-2.03
HFX_6171	PKD domain-containing protein		-2.04
HFX_1127	halocyanin domain-containing protein		-2.07
HFX_5092	plastocyanin/azurin family copper-binding protein		-2.27
HFX_2190	halocyanin domain-containing protein		-2.51
HFX_1128	Htur_1727 family rSAM-partnered candidate RiPP		-2.55
HFX_1340	halocyanin domain-containing protein		-2.80
