# Supplementary material for: Analysis of Lsm Protein-Mediated Regulation in the Haloarchaeon Haloferax mediterranei
Source: Int J Mol Sci. 2024 Jan 1;25(1):580. doi: 10.3390/ijms25010580 (PMC10779274; doi:10.3390/ijms25010580)
Supplement: Supplementary file 1 [file ijms-25-00580-s001.zip › Table S5.pdf]

Table S5. List of hypothetical genes *up-expressed* and *down-expressed* in the comparison between HM26 in the absence of a carbon source versus the absence of a nitrogen source. The table also includes the changes suffered in the name of some hypothetical proteins in NCBI on November 28.

Locus	Description	Log_2FC
HFX_6047	hypothetical protein - DUF1326 domain-containing protein	5.50
HFX_1737	hypothetical protein - DUF6789 family protein	4.58
HFX_2179	hypothetical protein - DUF2249 domain-containing protein	4.27
HFX_5251	hypothetical protein	3.60
HFX_6053	hypothetical protein - saccharopine dehydrogenase NADP-binding domain-containing protein	3.51
HFX_1389	hypothetical protein	3.30
HFX_1730	hypothetical protein	3.29
HFX_6035	hypothetical protein - EthD family reductase	3.27
HFX_6155	hypothetical protein	3.16
HFX_6016	hypothetical protein - Zn-ribbon domain-containing OB-fold protein	3.11
HFX_5250	hypothetical protein	2.99
HFX_RS12045	hypothetical protein	2.92
HFX_1771	hypothetical protein - UPF0058 family protein	2.88
HFX_1725	hypothetical protein	2.75
HFX_RS13105	hypothetical protein	2.64
HFX_RS06585	hypothetical protein	2.62
HFX_2425	hypothetical protein	2.60
HFX_2762	hypothetical protein	2.54
HFX_2884	hypothetical protein - YIP1 family protein	2.49
HFX_0491	hypothetical protein	2.44
HFX_2316	hypothetical protein - response regulator	2.44
HFX_2067	hypothetical protein	2.40
HFX_6021	hypothetical protein	2.39
HFX_0100	hypothetical protein	2.39
HFX_6440	hypothetical protein	2.25
HFX_0514	hypothetical protein	2.25
HFX_1678	hypothetical protein	2.20
HFX_1777	hypothetical protein - DUF2267 domain-containing protein	2.18
HFX_6008	hypothetical protein - MgtC/SapB family protein	2.13
HFX_0953	hypothetical protein	2.11
HFX_RS11730	hypothetical protein	2.11
HFX_2890	hypothetical protein	2.01
HFX_4091	hypothetical protein	2.01
HFX_1041	hypothetical protein - DUF1684 domain-containing protein	2.00
HFX_1888	hypothetical protein - DUF5806 family protein	-2.01
HFX_5090	hypothetical protein	-2.08
HFX_0016	hypothetical protein	-2.14
HFX_1949	hypothetical protein - BGTF surface domain-containing protein	-2.14

HFX_1338	hypothetical protein	-2.16
HFX_1540	hypothetical protein	-2.21
HFX_5244	hypothetical protein - DUF11 domain-containing protein	-2.22
HFX_0825	hypothetical protein	-2.22
HFX_2129	hypothetical protein	-2.24
HFX_1618	hypothetical protein	-2.25
HFX_0460	hypothetical protein	-2.31
HFX_0166	hypothetical protein	-2.42
HFX_0932	hypothetical protein - HTH domain-containing protein	-2.57
HFX_5219	hypothetical protein - PHA granule structural protein PhaP	-2.64
HFX_0427	hypothetical protein	-2.79
HFX_6062	hypothetical protein	-2.85
HFX_5218	hypothetical protein - AbrB/MazE/SpoVT family DNA-binding domain-containing protein	-2.87
HFX_1260	hypothetical protein	-2.97
HFX_0941	hypothetical protein	-3.00
HFX_1864	hypothetical protein - DUF4013 domain-containing protein	-3.08
HFX_1569	hypothetical protein	-3.09
HFX_1945	hypothetical protein	-3.30
HFX_5108	hypothetical protein	-3.37
HFX_0721	hypothetical protein - DoxX family membrane protein	-4.14
HFX_6343	hypothetical protein - DoxX family membrane protein	-4.27
HFX_RS04600	hypothetical protein	-4.33
HFX_1123	hypothetical protein	-5.00
HFX_0455	hypothetical protein - FmdB family zinc ribbon protein	-5.61
HFX_0454	hypothetical protein	-5.92
