# Supplementary material for: Analysis of Lsm Protein-Mediated Regulation in the Haloarchaeon Haloferax mediterranei
Source: Int J Mol Sci. 2024 Jan 1;25(1):580. doi: 10.3390/ijms25010580 (PMC10779274; doi:10.3390/ijms25010580)
Supplement: Supplementary file 1 [file ijms-25-00580-s001.zip › Table S6.pdf]

Table S6. List of *up-expressed* genes in the comparison between HM26 in the absence of a carbon source versus the absence of a nitrogen source.

Locus	Description	Log_2FC
**Genes related to carbon metabolism**		
HFX_2678	poly(R)-hydroxyalkanoic acid synthase subunit PhaC	3.87
HFX_0444	glyceraldehyde-3-phosphate dehydrogenase	3.81
HFX_6015	thiolase domain-containing protein	3.25
HFX_4010	glucose 1-dehydrogenase	3.25
HFX_1512	fructose-bisphosphatase class I	2.87
HFX_2242	aldehyde ferredoxin oxidoreductase family protein	2.64
HFX_6038	pdhA pyruvate dehydrogenase (acetyl-transferring) E1 component subunit alpha	2.53
HFX_6041	NAD-dependent succinate-semialdehyde dehydrogenase	2.49
HFX_2079	aceA isocitrate lyase	2.37
HFX_2078	aceB malate synthase	2.36
HFX_6033	NAD(P)-dependent oxidoreductase	2.03
**Genes related to amino acid metabolism**		
HFX_2865	aminotransferase	4.15
HFX_6378	1,2-phenylacetyl-CoA epoxidase subunit PaaB	3.36
HFX_6379	1,2-phenylacetyl-CoA epoxidase subunit PaaA	3.19
HFX_6329	urocanate hydratase	3.08
HFX_6377	1,2-phenylacetyl-CoA epoxidase subunit PaaC	3.08
HFX_6332	histidine ammonia-lyase	3.03
HFX_6331	imidazolonepropionase	2.76
HFX_6330	formimidoylglutamase	2.59
HFX_2939	PLP-dependent aspartate aminotransferase family protein	2.07
HFX_6192	creatininase family protein	2.06
**Genes related to energy metabolism**		
HFX_1733	cytochrome c oxidase subunit II	5.95
HFX_1732	cbb3-type cytochrome c oxidase subunit I	5.59
HFX_1731	cytochrome c oxidase subunit IV family protein	4.66
HFX_6014	superoxide dismutase	4.49
HFX_2688	FAD-binding oxidoreductase	2.48
HFX_5227	NAD(P)-dependent alcohol dehydrogenase	2.02
**Genes related to DNA metabolism and processing**		
HFX_1685	DNA starvation/stationary phase protection protein DpsA	5.70
HFX_0168	helix-turn-helix domain-containing protein	4.26
HFX_5171	restriction endonuclease	3.97
HFX_6439	HTH domain-containing protein	2.84
HFX_2930	5S ribosomal RNA	2.69
HFX_1280	TrmB family transcriptional regulator	2.40
HFX_2933	16S ribosomal RNA	2.37
HFX_6048	stress response translation initiation inhibitor YciH	2.03
HFX_4066	transcription initiation factor IIB family protein	2.03
HFX_6154	SprT-like domain-containing protein	2.02
HFX_6454	helix-turn-helix domain-containing protein	2.01

**Genes related to lipid metabolism**		
HFX_1281	beta-ketoacyl-ACP reductase	2.39
**Genes that encode transporters**		
HFX_6039	sodium-dependent transporter	3.80
HFX_6028	extracellular solute-binding protein	3.41
**Genes related to nitrogen metabolism**		
HFX_2178	glutamate dehydrogenase GdhB	7.26
HFX_1518	Glu/Leu/Phe/Val dehydrogenase	2.40
**Genes related to the processing of environmental information**		
HFX_6018	CBS domain-containing protein	2.78
HFX_6029	ATP-binding protein	2.65
