# Supplementary material for: Analysis of Lsm Protein-Mediated Regulation in the Haloarchaeon Haloferax mediterranei
Source: Int J Mol Sci. 2024 Jan 1;25(1):580. doi: 10.3390/ijms25010580 (PMC10779274; doi:10.3390/ijms25010580)
Supplement: Supplementary file 1 [file ijms-25-00580-s001.zip › Table S7.pdf]

Table S7. List of *down-expressed* genes in the comparison between HM26 in the absence of a carbon source versus the absence of a nitrogen source.

Locus	Description	Log_2FC
**Genes related to amino acid metabolism**		
HFX_0050	argininosuccinate synthase	-2.08
HFX_0041	ornithine carbamoyltransferase	-2.08
HFX_3015	O-acetylhomoserine aminocarboxypropyltransferase/cysteine synthase family protein	-2.08
HFX_2464	phosphoribosylanthranilate isomerase	-2.22
HFX_3012	O-acetylhomoserine aminocarboxypropyltransferase/cysteine synthase family protein	-2.29
HFX_1109	4-hydroxy-tetrahydrodipicolinate synthase	-2.32
HFX_0047	lysine biosynthesis protein LysX	-2.33
HFX_0045	N-acetyl-gamma-glutamyl-phosphate reductase	-2.37
HFX_0049	argininosuccinate lyase	-2.41
HFX_1108	4-hydroxy-tetrahydrodipicolinate reductase	-2.48
HFX_2754	5-methyltetrahydropteroyltriglutamate--homocysteine S-methyltransferase	-2.53
HFX_0747	tryptophan synthase subunit beta	-2.53
HFX_2463	anthranilate synthase component I	-2.85
HFX_1570	ketol-acid reductoisomerase	-2.97
HFX_1106	diaminopimelate decarboxylase	-3.01
HFX_6346	tryptophan synthase subunit beta	-3.03
HFX_0048	lysine biosynthesis protein LysW	-3.09
HFX_2753	methionine synthase	-3.28
HFX_0746	indole-3-glycerol phosphate synthase	-3.60
HFX_0748	tryptophan synthase subunit alpha	-3.89
**Genes that encode transporters**		
HFX_2451	EamA family transporter	-2.12
HFX_1205	ABC transporter ATP-binding protein	-2.12
HFX_1202	ABC transporter substrate-binding protein	-2.15
HFX_1204	branched-chain amino acid ABC transporter permease	-2.20
HFX_2706	carbohydrate ABC transporter permease	-2.24
HFX_2809	ABC transporter substrate-binding protein	-2.58
HFX_1788	L-lactate permease	-2.64
HFX_1831	ABC transporter substrate-binding protein	-2.70
HFX_1832	branched-chain amino acid ABC transporter permease	-2.73
HFX_2896	extracellular solute-binding protein	-2.87
HFX_2708	substrate-binding domain-containing protein	-2.90
HFX_2707	sugar ABC transporter permease	-3.24
HFX_1836	ATP-binding cassette domain-containing protein	-3.51
HFX_1834	ABC transporter ATP-binding protein	-3.73
HFX_2003	MFS transporter	-4.09
HFX_5153	DMT family transporter	-4.55
**Genes related to nitrogen metabolism**		
HFX_1918	ferredoxin-nitrite reductase	-2.10
HFX_6482	ferredoxin-nitrite reductase	-2.23
HFX_5091	TAT-dependent nitrous-oxide reductase	-2.53

Locus	Description	Log_2FC
HFX_0245	glnA type I glutamate--ammonia ligase	-2.92
HFX_2462	trpG anthranilate synthase component II	-3.10
HFX_2002	nasA assimilatory nitrate reductase NasA	-3.27
HFX_0093	ammonium transporter	-3.75
HFX_2005	ferredoxin-nitrite reductase	-4.66
HFX_0092	P-II family nitrogen regulator	-4.81
HFX_0094	P-II family nitrogen regulator	-4.88
HFX_0844	gltB glutamate synthase large subunit	-4.91
HFX_0095	ammonium transporter	-6.17
	**Genes related to carbon metabolism**	
HFX_0617	(R)-citramalate synthase	-2.13
HFX_1566	3-isopropylmalate dehydrogenase	-2.14
HFX_2145	nucleotide sugar dehydrogenase	-2.22
HFX_1371	2-oxoacid: acceptor oxidoreductase subunit alpha	-2.39
HFX_0749	2-amino-3,7-dideoxy-D-threo-hept-6-ulosonate synthase	-2.47
HFX_2637	phosphoenolpyruvate carboxylase	-2.59
HFX_1567	3-isopropylmalate dehydratase small subunit	-2.66
HFX_0519	C-terminal binding protein	-2.69
HFX_0958	aldo/keto reductase	-2.71
HFX_1568	3-isopropylmalate dehydratase large subunit	-3.04
HFX_1553	mandelate racemase/muconate lactonizing enzyme family protein	-3.14
HFX_1370	thiamine pyrophosphate-dependent enzyme	-3.19
HFX_1571	acetolactate synthase small subunit	-3.30
HFX_1572	biosynthetic-type acetolactate synthase large subunit	-3.60
HFX_1573	2-isopropylmalate synthase	-3.60
	**Genes related to DNA metabolism and processing**	
HFX_0640	aspartate--tRNA(Asn) ligase	-2.02
HFX_1893	bacterio-opsin activator domain-containing protein	-2.25
HFX_2941	phenylalanine--tRNA ligase subunit alpha	-2.27
HFX_1539	METTL5 family protein	-2.34
HFX_1341	helix-turn-helix domain-containing protein	-2.44
HFX_0819	helix-turn-helix domain-containing protein	-2.64
HFX_0959	helix-turn-helix domain-containing protein	-2.93
HFX_0165	winged helix-turn-helix transcriptional regulator	-3.53
HFX_0459	helix-turn-helix domain-containing protein	-3.95
HFX_0453	helix-turn-helix domain-containing protein	-4.54
HFX_0731	winged helix-turn-helix domain-containing protein	-4.61
HFX_1124	helix-turn-helix domain-containing protein	-4.91
	**Genes related to energy metabolism**	
HFX_1925	FAD-dependent oxidoreductase	-2.08
HFX_0565	2-phosphosulfolactate phosphatase	-2.14
HFX_2857	bifunctional methylenetetrahydrofolate dehydrogenase/methenyltetrahydrofolate cyclohydrolase FolD	-2.48
HFX_1926	electron transfer flavoprotein subunit alpha/FixB family protein	-2.66
HFX_0429	cytochrome ubiquinol oxidase subunit I	-2.67
HFX_0428	cytochrome d ubiquinol oxidase subunit II [	-3.12
HFX_1927	electron transfer flavoprotein subunit beta/FixA family protein	-3.12

Locus	Description	Log_2FC
HFX_0942	ba3-type terminal oxidase subunit CbaD	-3.13
HFX_0943	cytochrome c oxidase subunit II	-3.21
HFX_2214	molybdopterin-dependent oxidoreductase	-3.20
**Genes related to the metabolism of cofactors and vitamins**		
HFX_2322	bifunctional precorrin-2 dehydrogenase/sirohydrochlorin ferrochelatase	-2.01
HFX_2801	GTP 3',8-cyclase MoaA	-2.10
HFX_2004	molybdenum cofactor guanylyltransferase	-2.71
**Genes related to nucleotide metabolism**		
HFX_1619	phosphoribosylformylglycinamidine cyclo-ligase	-2.18
HFX_2246	phosphoribosylformylglycinamidine synthase I	-2.34
HFX_2251	phosphoribosylaminoimidazolesuccinocarboxamide synthase	-2.43
HFX_1046	phosphoribosylformylglycinamidine synthase subunit PurL	-2.50
HFX_1093	bifunctional phosphoribosylaminoimidazolecarboxamide formyltransferase/IMP cyclohydrolase	-2.54
**Genes that encode stress proteins**		
HFX_1094	Universal stress protein	-2.63
HFX_0946	Universal stress protein	-2.66
HFX_1885	Universal stress protein	-3.08
HFX_0369	Universal stress protein	-3.61
HFX_1928	Universal stress protein	-4.03
**Genes related to the processing of environmental information**		
HFX_1947	CBS domain-containing protein	-2.02
**Genes related to lipid metabolism**		
HFX_1837	long-chain-fatty-acid--CoA ligase	-2.69
**Genes related to signaling and cellular processes**		
HFX_6257	type IV pilin N-terminal domain-containing protein	-2.15
HFX_1895	PAS domain S-box protein	-2.55
HFX_1696	gas vesicle structural protein GvpA	-2.57
