# Supplementary material for: Analysis of Lsm Protein-Mediated Regulation in the Haloarchaeon Haloferax mediterranei
Source: Int J Mol Sci. 2024 Jan 1;25(1):580. doi: 10.3390/ijms25010580 (PMC10779274; doi:10.3390/ijms25010580)
Supplement: Supplementary file 1 [file ijms-25-00580-s001.zip › Table S8.pdf]

Table S8. Molecular exclusion chromatography results: standard proteins and Lsm protein.

	MW (kDa)	Log MW	Ve (ml)	Ve/Vo
**Standard proteins**				
Dextran blue	2000	3.30	37	1
β-amylase	200	2.30	44	1.12
Alcohol dehydrogenase	150	2.18	49	1.32
Albumin	66	1.82	55	1.49
Carbonic anhydrase	29	1.46	69	1.86
Cytochrome c	12.4	1.09	76	2.05
**Lsm protein**				
20 mM Tris, 0.5 M NaCl	**295**	2.47	40	1.08
	**27.5**	1.44	68	1.84
20 mM Tris. 1 M NaCl	**295**	2.47	40	1.08
20 mM Tris. 1.5 M NaCl	**588.8**	2.77	32	0.86
	**295**	2.47	40	1.08
20 mM Tris. 2 M NaCl	**588.8**	2.77	32	0.86
	**295**	2.47	40	1.08
