# Supplementary figures and images for: Analysis of Lsm Protein-Mediated Regulation in the Haloarchaeon Haloferax mediterranei
Source: Int J Mol Sci. 2024 Jan 1;25(1):580. doi: 10.3390/ijms25010580 (PMC10779274; doi:10.3390/ijms25010580)

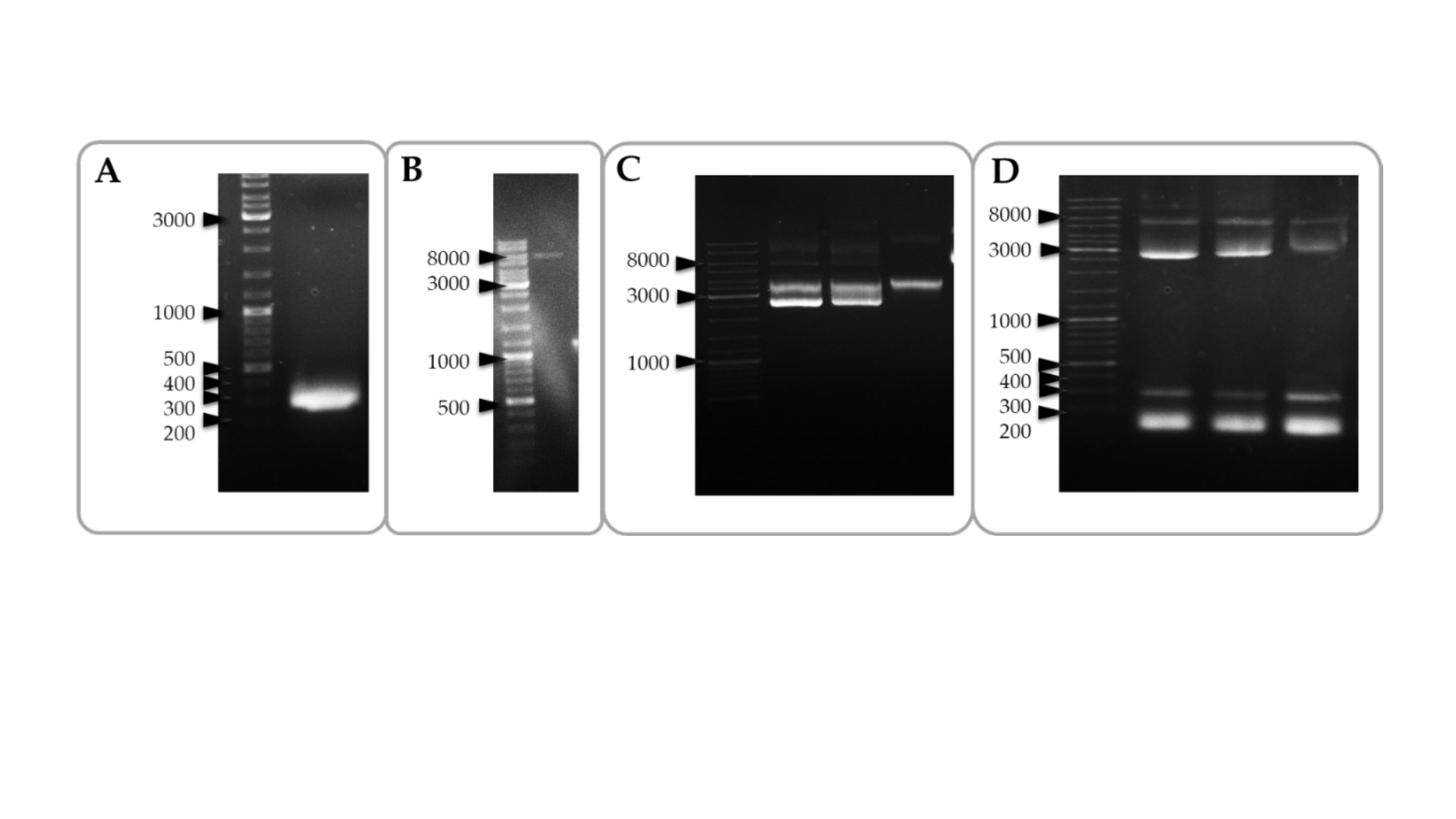

Supplement: Supplementary file 1 [file ijms-25-00580-s001.zip › Figure_S2.tif]

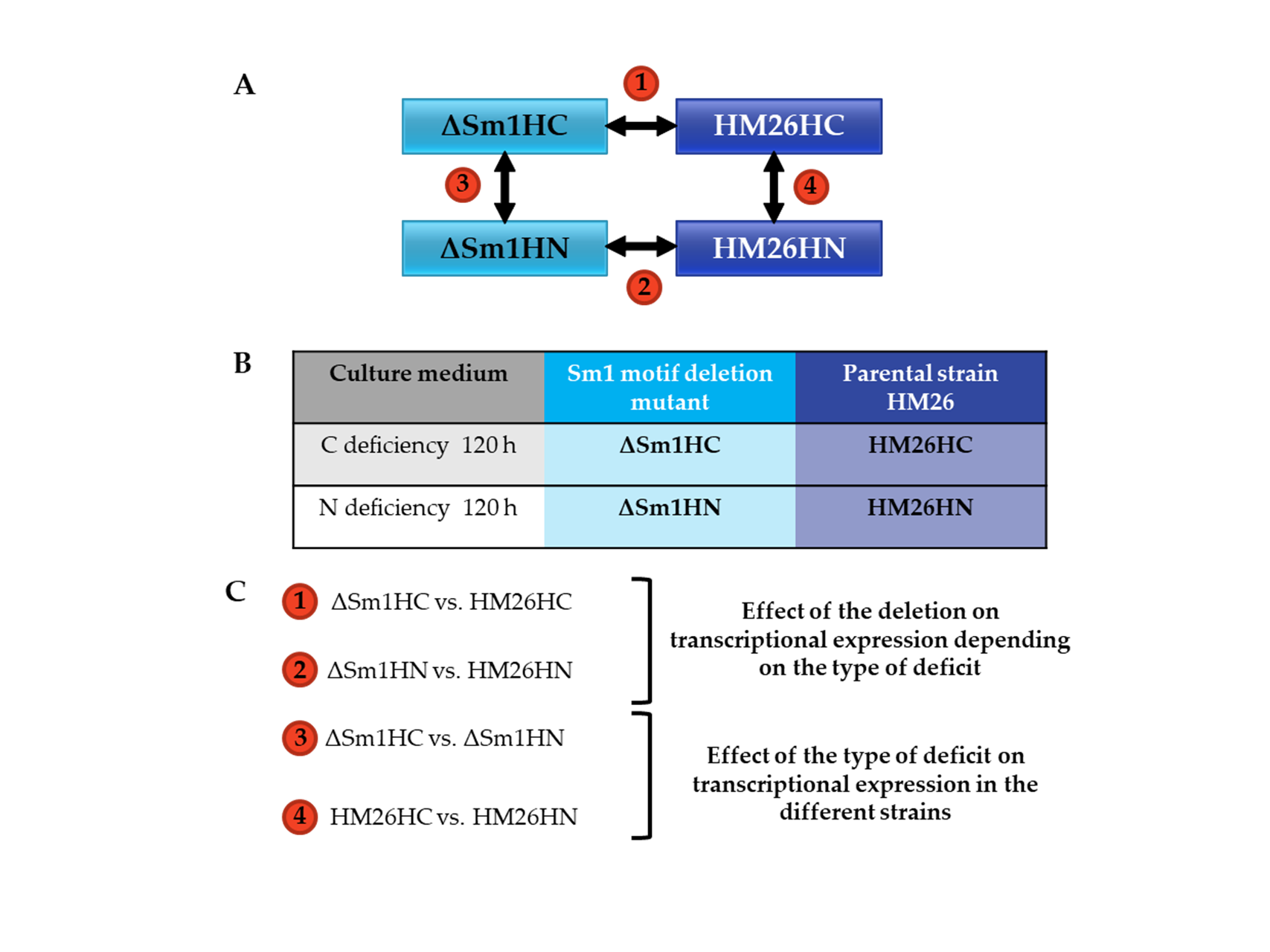

Supplement: Supplementary file 1 [file ijms-25-00580-s001.zip › Figure_S4.tif]
